# The Association between Antidepressant Medications and Coronary Heart Disease in Brazil: A Cross-Sectional Analysis on the Brazilian Longitudinal Study of Adult Health (ELSA-Brazil)

**DOI:** 10.3389/fpubh.2015.00009

**Published:** 2015-01-22

**Authors:** Andrew H. Kemp, Andre R. Brunoni, Marcio S. Bittencourt, Maria A. Nunes, Isabela M. Benseñor, Paulo A. Lotufo

**Affiliations:** ^1^Faculty of Medicine, University Hospital, University of São Paulo, São Paulo, Brazil; ^2^School of Psychology and Discipline of Psychiatry, University of Sydney, Sydney, NSW, Australia; ^3^Faculty of Medicine, Federal University of Rio Grande do Sul, Porto Alegre, Brazil

**Keywords:** tricyclic antidepressants, coronary heart disease, Brazil, cross-sectional design, clinical epidemiology, TCA, CHD

## Abstract

**Background:** Recent studies have highlighted associations between use of antidepressant medications and coronary heart disease (CHD). Tricyclic antidepressants (TCA) are not recommended in patients with CHD as they may increase morbidity and mortality. However, this class of antidepressants is freely prescribed in public health pharmacies, while access to other classes of antidepressants is restricted in Brazil. Here, we examine the associations between antidepressant use and prevalent CHD in a large cohort from Brazil.

**Methods:** Participants included 14,994 civil servants aged 35–74 years from the baseline assessment of the Brazilian Longitudinal Study of Adult Health (ELSA-Brasil). CHD (*n* = 710) included stable angina, myocardial infarction, and coronary revascularization. Univariate (unadjusted) and multivariate (adjusted) logistic regression analyses were conducted to estimate odds ratios and confidence intervals.

**Results:** After full adjustment for covariates, TCA use (*n* = 156) was associated with a twofold increase in prevalent CHD, relative to non-use (*n* = 14,076). Additional sensitivity analysis revealed a threefold association for myocardial infarction (OR: 2.96, 95% CI: 1.41–6.21) and coronary revascularization (OR: 2.92, 95% CI: 1.28–6.66). There were no significant associations between antidepressant use and stable angina pectoris.

**Conclusion:** Findings highlight a strong association between TCA use and prevalent CHD. While the cross-sectional design is an important limitation of the present study, findings have important implications for the treatment of cardiac patients in Brazil.

## Introduction

Coronary heart disease (CHD) and major depressive disorder (MDD) are leading burdens of disease (1) and the relationship between these disorders is bidirectional: patients with CHD have more MDD than the general population, while those with MDD are more likely to develop CHD (2, 3). Critically, comorbidity between CHD and MDD increases risk of further morbidity and mortality (4). Other studies have highlighted the association between CHD and the anxiety disorders (5, 6). Antidepressant use in patients with CHD is controversial. While use of tricyclic antidepressants (TCA) is not recommended (7), research indicates that all classes of antidepressant medications may have adverse cardiac effects (8, 9) [but see Ref. (10, 11) in regards to the selective serotonin reuptake inhibitor, or SSRI, antidepressant class]. The selective serotonin reuptake inhibitors are generally considered to be the safest class of antidepressants for use in cardiac patients (12) when needed. Here, we examine the association between antidepressant use and prevalent CHD in a large epidemiological cohort from Brazil.

The cardiovascular risks of TCA are well known (13). Although initially it was believed that TCAs could suppress arrhythmias in depressed patients with pre-existing arrhythmias, this belief was revised more than 20 years ago (13). The TCAs are also potent antagonists of muscarinic acetylcholine receptors (14) at the sinoatrial node of the heart leading to a decrease in parasympathetic activity, disinhibition of sympathetic nervous system activity, and tachycardia (15), which may lead to adverse cardiovascular events. While an earlier case–control study (16) reported that TCAs only increased the risk of myocardial infarction within the initial 28 days of antidepressant use, more recent research (17) demonstrated that use of TCAs are associated with a 35% increased risk of cardiovascular death over an 8-year follow-up period in initially healthy individuals. Consistent with this body of literature, a recent consensus statement from the National Heart Foundation of Australia (7), a high-income country, recommends that TCA be avoided in patients with CHD and depression.

While recommendations are helpful, they are difficult to apply in less-developed countries. Brazil is an upper-middle-income country facing major social challenges that may impact on the associations between CHD and antidepressant use. We have reported previously (18) that only 14 and 16.5% of patients in Brazil with generalized anxiety and MDD, respectively, take antidepressant medication. We also observed that while SSRIs were prescribed twice more frequently than tricyclic medications, antidepressant use was related to having private health insurance. TCA are freely dispensed in public health pharmacies in Brazil, while most of the SSRI medications are not, with the exception of fluoxetine and, in some regions of Brazil including São Paulo, sertraline (18, 19). (The list of medicines supplied by the Brazilian Unified Health System, or SUS, in São Paulo is available here: http://www2.hu.usp.br/confira-lista-de-medicamentos-do-sus/.) TCA therefore play an important role in treating depression, as well as a variety of other conditions including neuropathic pain and fibromyalgia (20) in Brazil. This context provides an important background for the current study, which sought to determine the associations between use of antidepressant medications and prevalent CHD in the Brazilian Longitudinal Study of Adult Health (ELSA-Brasil) (21, 22) cohort. We examine associations in the cohort at baseline to provide an important foundation for future prospective analyses on this cohort.

## Materials and Methods

### Participants

ELSA-Brasil is a cohort of 15,105 civil servants aged 35–74 years enrolled between August 2008 and December 2010 at six cities (Belo Horizonte, Porto Alegre, Rio de Janeiro, Salvador, Sao Paulo, and Vitoria) designed to investigate the relationship between cardiovascular diseases and diabetes, their social determinants, and risk factors. The study design and sampling procedures of ELSA-Brasil have been reported previously (21, 22). A total of 14,994 participants are reported here after dropping a relatively small number of cases (*n* = 111) with missing data on variables that were included in analyses.

### Ethics statement

The ethics committees of the participating universities as well as the National Research Ethics Committee approved the research protocol. All participants provided written informed consent after a complete description of the study.

### Psychiatric evaluation

Mental disorders were determined by trained interviewers using the Portuguese version (23) of the Clinical Interview Schedule-Revised (CIS-R) (24). This is a structured interview used for diagnosis of common, non-psychotic psychiatric conditions in the community. The complete CIS-R version was applied, severity scores were obtained, and common mental disorder status (CIS-R scores ≥12), determined. Antidepressant medications [Anatomical Therapeutic Chemical (ATC) Classification code: N06A] included the selective serotonin reuptake inhibitors (SSRI, ATC code: N06AB), the serotonin and noradrenaline reuptake inhibitors (SNRIs, ATC codes: N06AX16/N06AX23/N06AX21), the TCA (ATC code: N06AA), and other antidepressants (N06AX22, N06AX12, N06AA21, N06AX05, N06AX11). Individuals taking at least one antidepressant medicine continuously in the past 2 weeks were classified as users.

### Coronary heart disease assessment

Coronary heart disease (CHD) included participants with stable angina pectoris, myocardial infarction, and coronary revascularization determined through questionnaire- and intensive interview-based assessment focusing on medical history. All prior CHD were self-referred by the patients during the structured interview. For the current analysis, prevalent CHD was defined as a prior history of a physician diagnosed myocardial infarction, a prior percutaneous coronary intervention including balloon angioplasty with or without stent placement, a prior surgical revascularization consisting of either arterial or venous grafts and the history of stable angina as defined by a physician taking care of the participant prior to the inclusion in the ELSA study. The outcome of coronary revascularization was defined as either a percutaneous coronary intervention or a surgical revascularization as previously described.

### Statistical analysis

Statistical analysis was conducted using IBM SPSS Statistics Version 21. Participant characteristics were examined using independent samples *t*-tests and one-way analyses of variance (ANOVA) for contrasts involving continuous dependent measures, and χ^2^ statistics for categorical variables. Degrees of freedom were corrected when Levene’s Test for Equality of Variances was violated. Tukey’s HSD is reported to correct for multiple comparisons and aid interpretation of ANOVA’s, while standardized residuals (*z*-scores) were used to help interpret chi-square tests on larger contingency tables [as per Ref. (25)].

A series of univariate and multivariate, binary, logistic regression analyses were then used to estimate the odds ratios and 95% confidence intervals for the association between antidepressant use as the independent variable (IV) and prevalent CHD (no, yes) as the dependent variable (DV), before and after adjustment for covariates. Unadjusted (univariate) and adjusted (multivariate) analyses were also conducted on specific classes of antidepressants (IV) including the SSRIs, the SNRIs, tricyclic medications and others, and prevalent CHD (no, yes) (DV). Unadjusted univariate, binary, logistic regression analyses (model 1) were conducted on antidepressant use (no versus yes), as well as classes of antidepressants, with no other predictors. Adjusted multivariate analyses (model 2) involved binary logistic regression analysis in which covariates were entered into the first block using the enter method, and antidepressant use was entered into the second (final) block, a technique known as sequential logistic regression analysis. Covariates included age, sex, education (less than high-school, high-school, university), smoking status (never, past/current), body mass index (BMI; weight in kilograms divided by height in meters squared), hypertension (systolic blood pressure ≥140 mm Hg, or diastolic blood pressure ≥90 mm Hg, or use of antihypertensive medications), diabetes mellitus (DM) (self-reported or fasting blood glucose ≥126 mg/dL, 2-h oral glucose tolerance test ≥200 mg/dL, or glycated hemoglobin ≥6.5%), and severity of mood and anxiety disorders. Sequential, logistic regression analysis is a common approach that allows for the independent contribution of antidepressant use over and above covariates to be determined (26). Statistics from multivariate analyses are reported for the overall model and block (after adjusting for covariates). The block statistic indicates whether or not the IV of interest (antidepressant use/class) is significantly associated with the dependent measure, CHD. Sensitivity analyses were conducted for specific CHD events to determine the consistency of associations across distinct categories of CHD. Sensitivity analyses involved sequential, binary logistic regression analysis in which covariates were entered into block 1 using the enter method, followed by antidepressant use in block 2 (as per model 2).

## Results

### Participant characteristics

Table 1 summarizes participant characteristics by CHD status, while Table 2 presents participant characteristics according to antidepressant use. Prevalent CHD was characterized by older age, more men, less education, more smokers, higher BMI, more individuals with DM, hypertension and common mental disorder, higher CIS-R score, and antidepressant use. Antidepressant use was characterized by older age, more women, more education, more smokers, more individuals with CHD and common mental disorder, and higher CIS-R score. Table 3 provides a more detailed breakdown of participant characteristics by antidepressant class. Notably, TCA use is characterized by more women, fewer individuals with college-level education and more with CHD and common mental disorder, and a higher CIS-R score.

**Table 1:** **Participant characteristics by CHD status (*N* = 14,994)**.

Characteristics	No CHD (*n* = 14,284, 95%)	CHD (*n* = 710, 5%)	Statistic
Age, mean (SD)	51.75 (8.97)	58.66 (8.74)	*t*(14,992) = 20.07, *p* < 0.001
Women (%)	54.8	45.5	χ^2^(1) = 23.45, *p* < 0.001
Education (%)			χ^2^(2) = 85.16, *p* < 0.001
Less than high-school	12.1	23.7*	
High-school	34.8	33.8	
College	53.1	42.5*	
Smoker (past or current) (%)	42.5	56.1	χ^2^(1) = 50.77, *p* < 0.001
Body mass index (kg/m^2^), mean (SD)	26.95 (4.74)	28.37 (4.80)	*t*(14,992) = 7.76, *p* < 0.001
Diabetes mellitus (yes) (%)	18.7	37.9	χ^2^(1) = 157.21, *p* < 0.001
Hypertension (yes) (%)	34.0	70.0	χ^2^(1) = 382.49, *p* < 0.001
Common mental disorder (yes) (%)	26.2	35.5	χ^2^(1) = 29.86, *p* < 0.001
CIS-R Score, mean (SD)	8.08 (7.86)	10.34 (9.61)	*t*(756.82) = 6.18, *p* < 0.001
Antidepressant use (yes) (%)	6.0	8.6	χ^2^(1) = 7.91, *p* = 0.005

CIS-R, Clinical Interview Schedule-Revised. *Refers to categories with standardized residuals (*z*-scores) lying outside ± 1.96 reflecting a significance value of *p* < 0.05.

**Table 2:** **Participant characteristics by antidepressant use (*N* = 14,994)**.

Characteristics	No (*n* = 14,076, 94%)	Yes (*n* = 918, 6%)	*p*-Value
Age, mean (SD)	51.97 (9.07)	53.70 (9.14)	*t*(14,992) = 5.59, *p* < 0.001
Women (%)	52.9	76.4	χ^2^(1) = 191.32, *p* < 0.001
Education (%)			χ^2^(2) = 34.90, *p* < 0.001
Less than high-school	13.0	7.8*	
High-school	35.0	31.2	
College	52.0	61.0*	
Smoker (past or current) (%)	42.9	47.1	χ^2^(1) = 6.15, *p* = 0.013
Body mass index (kg/m^2^), mean (SD)	27.01 (4.75)	27.09 (4.76)	*t*(14,992) = 0.48, *p* = 0.632
Diabetes mellitus (yes) (%)	19.8	17.8	χ^2^(1) = 2.20, *p* = 0.138
Hypertension (yes) (%)	35.6	36.5	χ^2^(1) = 0.28, *p* = 0.596
CHD status (yes) (%)	4.6	6.7	χ^2^(1) = 8.39, *p* = 0.004
Common mental disorder (yes) (%)	25.4	45.6	χ^2^(1) = 180.59, *p* < 0.001
CIS-R Score, mean (SD)	7.92 (7.75)	12.27 (9.90)	*t*(991.68) = 13.06, *p* < 0.001

CIS-R, Clinical Interview Schedule-Revised. *Refers to categories with standardized residuals (*z*-scores) lying outside ± 1.96 reflecting a significance value of *p* < 0.05.

**Table 3:** **Participant characteristics by antidepressant grouping (*N* = 14,994)**.

Characteristics	CTL (*n* = 14,076)	SSRI (*n* = 567)	SNRI (*n* = 100)	TCA (*n* = 156)	Other (*n* = 95)	*p-*Value
Age, mean (SD)	51.97 (9.07)	53.70* (9.36)	55.48* (8.60)	53.42 (8.45)	52.26 (9.33)	*F*(4,14,993) = 9.41, *p* < 0.001
Women,%	52.9*	77.8*	81.0*	75.0*	65.3	χ^2^(4) = 197.48, *p* < 0.001
Education (%)						χ^2^(8) = 80.32, *p* < 0.001
Less than high-school	13.0	6.7*	4.0*	15.4	6.3
High-school	35.0	28.6*	23.0*	47.4*	28.4	
College	52.0	64.7*	73.0*	37.2*	65.3	
Smoker (past or current) (%)	42.9	47.1	40.0	47.4	53.7	χ^2^(4) = 9.89, *p* = 0.042
BMI (kg/m^2^), mean (SD)	27.01 (4.75)	27.12 (4.89)	27.68 (4.62)	26.83 (4.06)	26.69 (5.21)	*F*(4,14,993) = 0.731, *p* = 0.571
Hypertension (yes)%	35.6	34.9	45.0	38.5	33.7	χ^2^(4) = 4.64, *p* = 0.33
Diabetes (yes)%	19.8	17.3	18.0	20.5	15.8	χ^2^(4) = 3.27, *p* = 0.51
CHD (yes) (%)	4.6	6.3	3.0	10.9*	5.3	χ^2^(4) = 17.61, *p* = 0.001
Common mental disorder (yes) (%)	25.4*	45.0*	43.0*	46.2*	51.6*	χ^2^(4) = 182.81, *p* < 0.001
CIS-R Score, mean (SD)	7.92 (7.75)	12.17 (9.70)*	12.19 (10.51)*	12.48 (10.24)*	12.55 (9.94)*	*F*(4,14,993) = 65.42, *p* < 0.001

*Refers to one-way ANOVA in which each group is compared to controls (Tukey’s HSD, *p* < 0.05) or standardized residuals (*z*-scores) from χ^2^ statistics lying outside ± 1.96 reflecting a significance value of *p* < 0.05.

### Association between antidepressant use and prevalent CHD

Table 4 describes results of analyses assessing the association of antidepressant medications with prevalent CHD. Model 1 relates to the results of unadjusted analyses for antidepressant use [model χ^2^(1) = 7.12, *p* = 0.008] and antidepressant class [model χ^2^(4) = 14.03, *p* = 0.007], while model 2 relates to results adjusted for covariates [antidepressant use, model χ^2^(10) = 724.85, *p* < 0.001; block χ^2^(1) = 2.69, *p* = 0.101; antidepressant class, model χ^2^(10) = 732.04, *p* < 0.001; block χ^2^(4) = 9.88, *p* = 0.042]. Antidepressant use was associated with a 1.5-fold increase in the odds of prevalent CHD (95% CI: 1.12–1.93) (model 1), although this was reduced to a 1.3-fold increase when adjusting for covariates (95% CI: 0.96–1.71) (model 2). Sensitivity analysis revealed that use of TCAs (OR = 2.53, 95% CI: 1.52–4.21, model 1; OR = 2.15, 95% CI = 1.24–3.71) in particular is significantly associated with prevalent CHD.

**Table 4:** **Unadjusted (model 1)^a^ and adjusted (model 2)^b^ associations between antidepressant use and CHD (*N* = 14,994)**.

Predictor	*N*	CHD: model 1^a^ (*n* = 710)			*N*	CHD: model 2^b^ (*n* = 710)		
		OR	95% CI	*p*-Value		OR	95% CI	*p*-Value
Any antidepressant use								
No	14,076		REF		14,076		REF	
Yes	918	1.47	1.12–1.93	0.005	918	1.28	0.96–1.71	0.093
Antidepressant groupings								
None	14,076		REF		14,076		REF	
SSRI	567	1.40	0.99–1.98	0.056	567	1.26	0.87–1.81	0.218
SNRI	100	0.64	0.20–2.02	0.447	100	0.47	0.15–1.52	0.209
TCA	156	2.53	1.52–4.21	<0.001	156	2.15	1.24–3.71	0.006
Other	95	1.15	0.47–2.84	0.763	95	1.03	0.40–2.65	0.949

*^a^Model 1 relates to separate unadjusted analyses for any antidepressant use and specific classes of antidepressant including SSRI (selective serotonin reuptake inhibitors), SNRI (serotonin and noradrenaline reuptake inhibitors), TCA (tricyclic antidepressants), and other*.

*^b^Model 2 relates to analyses adjusted for covariates*.

### Association between antidepressant use and CHD subtypes

Table 5 reports results for the additional specificity analyses on stable angina pectoris [antidepressant use: model χ^2^(10) = 223.26, *p* < 0.001; block χ^2^(1) = 0.02, *p* = 0.891; antidepressant class: model χ^2^(13) = 223.26, *p* < 0.001; block χ^2^(4) = 3.02, *p* = 0.555], myocardial infarction [antidepressant use: model χ^2^(10) = 426.90, *p* < 0.001; block χ^2^(1) = 0.83, *p* = 0.361; antidepressant class: model χ^2^(13) = 437.14, *p* < 0.001; block χ^2^(4) = 11.07, *p* = 0.026], and coronary revascularization [antidepressant use: model χ^2^(10) = 440.68, *p* < 0.001; block χ^2^(1) = 6.02, *p* = 0.014; antidepressant class: model χ^2^(13) = 448.41, *p* < 0.001; block χ^2^(4) = 13.75, *p* = 0.008]. While there were no significant associations observed for stable angina, use of tricyclic medications were associated with a threefold increase in odds for myocardial infarction as well as coronary revascularization. These additional findings indicate that the association between TCA use and prevalent CHD is specific to “hard” CHD events including myocardial infarction and coronary revascularization.

**Table 5:** **Fully adjusted association between antidepressant use and CHD subtypes (*N* = 14,994)**.

Predictor	*N*	Stable angina (*n* = 312)^a^			*N*	Myocardial infarction (*n* = 267)			*N*	Coronary revascularization (*n* = 255)		
		OR	95% CI	*p*-Value		OR	95% CI	*p*-Value		OR	95% CI	*p*-Value
Any antidepressant use								
No	13,715			REF	14,076			REF	14,076			REF
Yes	881	0.85	0.54–1.36	0.501	918	1.25	0.78–2.01	0.349	918	1.79	1.15–2.77	0.009
Antidepressant groupings								
None	13,715				14,076			REF	14,076		REF	
SSRI	546	1.00	0.58–1.71	0.989	567	1.12	0.59–2.10	0.735	567	1.96	1.16–3.34	0.013
SNRI	98	0.30	0.04–2.16	0.229	100	n/a	n/a	0.996	100	1.15	0.27–4.85	0.845
TCA	143	1.01	0.37–2.79	0.986	156	2.96	1.41–6.21	0.004	156	2.92	1.28–6.66	0.011
Other	94	1.65	0.59–4.64	0.340	95	0.54	0.07–4.03	0.547	95	n/a	n/a	0.996

*^a^This model excludes 398 participants with myocardial infarction or coronary revascularization*.

## Discussion

The goal of this study was to determine the associations between use of antidepressant medications and prevalent CHD in a cohort of civil servants from Brazil. This is an important goal because Brazil currently faces many socioeconomic inequities, which may impact on antidepressant usage. Major findings indicate that use of TCA is associated with: (1) a twofold increase in the odds for prevalent CHD and (2), a threefold increase in the odds for myocardial infarction and coronary revascularization, after adjustment for covariates. The associated 95% confidence intervals for TCA use – all of which excluded the null value of 1 – provide sets of likely values for the odds ratio on which for a repeated study would most likely fall (on average, a five-in-six chance) (27). Values close to the sample estimates, however, are ~7 times more likely to reflect the true population estimate (μ), than values near the limits of the interval (27). These considerations and the size of the effects obtained, enhance our confidence in the reported findings reported here.

While our study highlights a strong relationship between TCA use and “hard” CHD events, it is important to acknowledge that some participants in our study may have been using low-dose TCAs to treat conditions other than mental disorders, such as chronic/neuropathic pain and sleep issues. Importantly, research has demonstrated a dose-related increase in sudden cardiac death in current users of TCAs from 0.97 for doses lower than 100 mg (amitriptyline or its equivalent) to 2.53 for doses of 300 mg or more, highlighting that doses of <100 mg does not increase risk (at least for sudden cardiac death). However, current recommendations indicate that TCAs should be avoided completely in cardiac patients (7). In Brazil, access to TCAs is free (18, 19), while access to other classes of antidepressants is restricted suggesting socioeconomic reasons that may increase the association between TCA and prevalent CHD. It is notable that elderly Brazilian patients with psychiatric disorders are 5.3 times more likely to be using inappropriate medications (28). We suggest that these previous findings (28) may help to understand the findings that we report here, which may indicate problems associated with ongoing health care of cardiac patients in the Brazilian population.

It is important to acknowledge the cross-sectional design as an important limitation of the present study. This limitation precludes any conclusions over the causal relationship between TCA use and CHD. For instance, it is equally possible that TCA use preceded the development of CHD consistent with research that suggests TCAs lead to cardiovascular events [e.g., Ref. (17)] (i.e., a biological explanation) beyond that explained by psychiatric illness, or that TCAs were prescribed after CHD was diagnosed consistent with research that suggests patients may be inappropriately medicated in Brazil [e.g., Ref. (28)] (i.e., a sociodemographic explanation). However, regardless of the causal direction of the relationship between TCA use and CHD, our findings still have important implications for the treatment of cardiac patients in Brazil. It is notable here that research from the Netherlands (5), a high-income country, did not observe a significant association between use of TCAs and CHD. The authors noted that while the adverse cardiovascular effects of TCAs are well known, a null finding might reflect the contraindication of TCA use in heart patients. We suggest here that socioeconomic inequities in Brazil may over-ride recommendations to avoid these medications in cardiac patients as patients have easier and free access to this class of antidepressant medications through free public health care.

Longitudinal research (8) from the Netherlands Study of Depression and Anxiety (*N* = 2,114) indicates that all classes of antidepressants may have adverse effects on heart function, determined by reductions in heart rate variability, a psychophysiological predictor of future cardiovascular mortality [see in Ref. (29) for review]. Adverse effects were greatest for the TCAs, followed by the SNRIs, and then the SSRIs, relative to no antidepressant use (8) [see also in Ref. (30)]. This study also reported that these effects disappear when antidepressants are discontinued. However, the adverse effects reported for the SSRI class were small, which may, in part, explain the contradictory findings reported previously for the association between SSRIs and CHD (9, 17). SSRI antidepressants are generally considered to have a more favorable cardiovascular profile than the TCA (and the serotonin and noradrenaline reuptake inhibitors, or SNRIs). The SSRIs may exert cardiovascular benefits through direct action on the biological substrates of the stress response (31), including a blunting of blood pressure, myocardial responses, and cortisol reactivity under stress. The SSRIs also have antiplatelet properties, which will reduce the risk for thrombus formation (2, 3). While we did not observe a relationship between SSRIs and prevalent CHD, we did observe a twofold increase association for SSRIs and coronary revascularization. A possible explanation for the null association between SSRIs and myocardial infarction is the restricted access to antidepressants from the SSRI class of antidepressants, resulting perhaps from the socioeconomic inequities of Brazilian mental health care (28, 32, 33) and high cost of these newer medications.

In conclusion, the present study provides important new information on the association of antidepressant use and prevalent CHD in Brazil. While a limitation of our study is its cross-sectional design, this limitation does not undermine the importance of our findings, as TCA use in patients with CHD increases risk of future morbidity and mortality. While it is possible that some of our participants were on low-dose TCAs for conditions other than depression and anxiety, recommendations from high-income countries suggest that these medications should be avoided in cardiac patients. Our study is characterized by a number of strengths including a focus on a relatively large and well-characterized sample of the Brazilian population, application of a structured clinical interview to determine psychiatric diagnosis and disorder severity, and adjustment for a host of covariates known to contribute to metabolic and cardiovascular risk. Our findings indicate a strong relationship between TCA use and prevalent CHD. We will further examine the impact of the different antidepressant classes in a longitudinal follow-up study of the ELSA-Brasil cohort once data collection is complete.

## Author Contributions

Andrew H. Kemp conducted the literature search, identified the research questions, and clarified the hypotheses. He analyzed the data, interpreted the results, and wrote the paper. Maria A. Nunes adapted the Clinical Interview Schedule for use in our project and was responsible for the psychiatric evaluations of participants recruited in ELSA-Brasil. Isabela M. Bensen or and Paulo A. Lotufo have been involved in the ELSA-Brasil project since its inception, and secured the funding to initiate and conduct the project. They were involved in all aspects of the present study including research design, data collection, analysis, and interpretation of results. All authors reviewed and approved the manuscript for publication.

## Conflict of Interest Statement

The authors declare that the research was conducted in the absence of any commercial or financial relationships that could be construed as a potential conflict of interest.
